# What is said about #donateliver or #liverdonor? Reflexive thematic analysis of Twitter (X) posts from 2012 to 2022

**DOI:** 10.1186/s12889-024-19381-1

**Published:** 2024-07-16

**Authors:** Qin Xiang Ng, Yu Liang Lim, Xiaohui Xin, Clarence Ong, Wee Khoon Ng, Julian Thumboo, Hiang Khoon Tan

**Affiliations:** 1 Saw Swee Hock School of Public Health, National University of Singapore and National University Health System, Singapore, Singapore; 2 Health Services Research Unit, Singapore General Hospital, Singapore, Singapore; 3 Department of Gastroenterology and Hepatology, Tan Tock Seng Hospital, Singapore, Singapore; 4 SingHealth Duke-NUS Medicine Academic Clinical Programme, Duke-NUS Medical School, Singapore, Singapore; 5 Division of Surgery and Surgical Oncology, Singapore General Hospital and National Cancer Centre Singapore, Singapore, Singapore; 6 SingHealth Duke-NUS Global Health Institute, Singapore, Singapore; 7 Duke Global Health Institute, Duke University, Durham, USA

**Keywords:** Living donor, Liver donor, Twitter, X, Social media, Qualitative study

## Abstract

**Background:**

There is sustained interest in understanding the perspectives of liver transplant recipients and living donors, with several qualitative studies shedding light on this emotionally charged subject. However, these studies have relied primarily on traditional semi-structured interviews, which, while valuable, come with inherent limitations. Consequently, there remains a gap in our comprehension of the broader public discourse surrounding living liver donation. This study aims to bridge this gap by delving into public conversations related to living liver donation through a qualitative analysis of Twitter (now X) posts, offering a fresh perspective on this critical issue.

**Methods:**

To compile a comprehensive dataset, we extracted original tweets containing the hashtags “#donateliver” OR “#liverdonor”, all posted in English from January 1, 2012, to December 31, 2022. We then selected tweets from individual users whose Twitter (X) accounts featured authentic human names, ensuring the credibility of our data. Employing Braun and Clarke’s reflexive thematic analysis approach, the study investigators read and analysed the included tweets, identifying two main themes and six subthemes. The Health Policy Triangle framework was applied to understand the roles of different stakeholders involved in the discourse and suggest areas for policy improvement.

**Results:**

A total of 361 unique tweets from individual users were analysed. The major theme that emerged was the persistent shortage of liver donors, underscoring the desperation faced by individuals in need of life-saving liver transplants and the urgency of addressing the organ shortage problem. The second theme delved into the experiences of liver donors post-surgery, shedding light on a variety of aspects related to the transplantation process, including the visibility of surgical scars, and the significance of returning to physical activity and exercise post-surgery.

**Conclusion:**

The multifaceted experiences of individuals involved in the transplantation process, both recipients and donors, should be further studied in our efforts to improve the critical shortage of liver donors.

## Introduction

Globally, liver cirrhosis is one of the leading causes of mortality and morbidity [1]. Rates of liver failure continue to rise [2], and for patients with end-stage liver failure and advanced cirrhosis, liver transplantation remains the only casually directed treatment option that improves survival and quality of life [3]. However, the dramatic shortage of donor organs worldwide has been a consistent challenge, which results in long average wait times for a suitable organ donor [4], repeated changes in organ donation legislature and allocation methods [5], and transplant centers resorting to livers of extended criteria donors for transplantation [6]. 

In terms of options for liver transplantation, living donor transplantation appears to yield better perioperative outcomes and long-term survival than deceased donor transplantation [7]. Given that the liver has a remarkable ability to regenerate itself, coupled with the continued shortage of cadaveric donor organs, many countries have adopted legislative changes and initiated public campaigns with varying degrees of success in encouraging organ donation [8]. Previous qualitative studies have reported apprehension of donors owing to the tedious screening processes, lengthy preoperative assessment and potential physical and medical sequalae after surgery [9, 10]. Although prior qualitative studies have elucidated the lived experiences of transplant recipients and living donors, there are undoubtedly limitations with traditional semi-structured interviews and gaps in understanding with regard to a potential donor’s decision-making process and post-donation health management experience.

Twitter (recently renamed X), a popular social media platform with more than 250 million users worldwide, allows individuals and organizations to post and share short text-based messages (or “tweets”) with a large public audience [11]. These tweets (limited to 280 characters) are a form of microblogging that have been studied for sentiment analysis and public perception research [12, 13]. In recent years, there is also an increasing trend of patients and families turning to these platforms to crowdsource for potential suitable liver donors and organize online crowdfunding to support the medical costs of the procedure [14, 15]. Users have spontaneity and great flexibility to response to a topic or tweet of interest, which facilitates the analysis of the sentiments of a larger sample. Additionally, in contexts where organ donation remains a taboo subject [16], these platforms offer a unique opportunity for open discussion. This creates a valuable avenue for gaining new insights into the complicated and weighty issue of solid organ donation by examining public conversations on Twitter (X).

To guide the study, we apply the Health Policy Triangle (HPT) framework, developed by Walt and Gilson in 1994 [17]. The framework has been frequently applied to analyse health-related issues and concerns, especially among low/middle-income countries [18]. In our study, the HPT can provide insights into not only the content of the discussions but also the broader context, and the roles of various stakeholders in the discourse, thereby identifying potential areas for policy intervention and improvement.

## Methods

### Extraction of tweets

The methodology for the present exploratory study was adapted from previous infodemiology studies that also utilised Twitter (X) to investigate public perceptions and manifested emotions on a particular topic [12, 13]. We extracted original tweets containing the search terms “#donateliver” OR “#liverdonor”, using Twitter (X)’s Application Programming Interface (API) platform (using an academic developer account), and posted in the English language from 1 January 2012 to 31 December 2022. Other search terms were tried, including “organ donor”, “liver donee” and “living donor”, however, these appeared to identify non-related tweets and content, e.g. kidney transplant recipients and organ replacement in pet animals. We also tried to incorporate Boolean operators, for example, we used combinations like “liver AND donor”. However, this appeared to introduce more noise and irrelevant posts. Thus, we opted to focus on the primary hashtags to maintain the quality and relevance of our collected tweets. We also had to keep the total number of tweets manageable for manual thematic analysis. Retweets and duplicate tweets (i.e., tweets with identical sentences and words) were excluded from analysis. Tweets by organizations (e.g. agencies, news outlets and businesses) were excluded from analysis. Only tweets by individual users were included for analysis, and they were manually identified by the use of actual human names for the Twitter (X) account of each post.

### Qualitative analysis of tweets

Reflexive thematic analysis, as guided by the procedure outlined by Braun and Clarke [19], was then performed inductively by the study investigators. Thematic analysis was chosen in favour of content analysis as it has theoretical flexibility, provides a detailed and nuanced analysis, and is useful for studying individual experiences, opinions, and views [20]. By reading and re-reading the included tweets, the study investigators familiarized themselves with the data, produced preliminary codes, formulated overarching themes, reviewed and refined themes, defined and specified themes, and produced a write-up [19]. Study investigators reviewed the tweets independently, and coding disagreements were resolved by further discussion until consensus was reached. The study investigators also moved back and forth between the different steps during the analysis in an iterative manner.

### Ethical considerations

This study did not directly involve any human participants. All data used in the present study were collected according to Twitter (X)’s terms of use [11]. Additionally, to protect the anonymity of the post authors, potentially sensitive information, their account IDs and any references to other account IDs (e.g., the use of @) were removed.

## Results

**Fig. 1:**
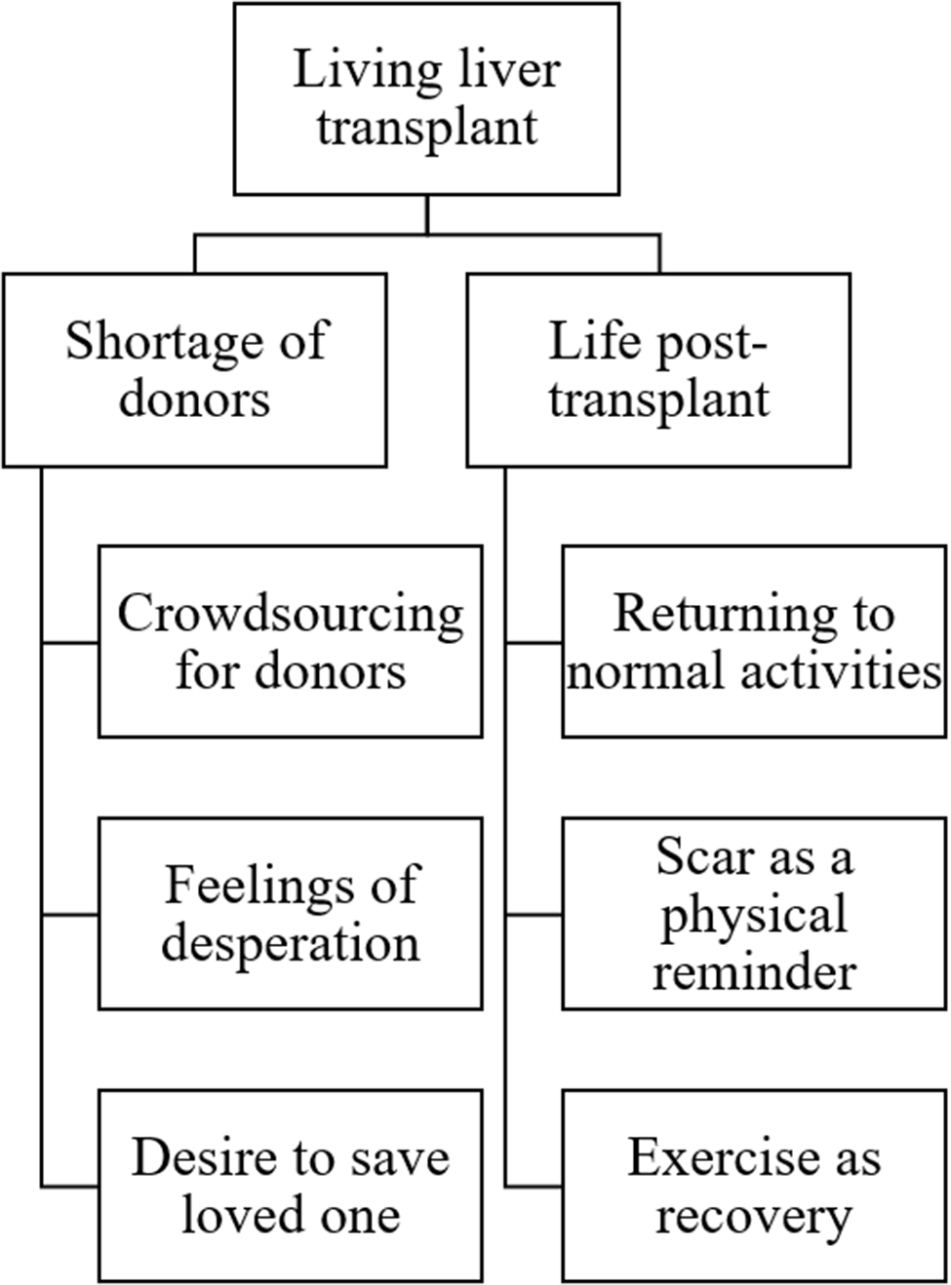
Thematic map of themes and subthemes identified

A total of 517 unique, English-language tweets were extracted. Of these, 156 tweets were posted by organisations over Twitter (X), leaving 361 unique tweets from individual users for analysis. Two themes and six subthemes are reported alongside sample tweets (Table 1) and a thematic map (Fig. 1).

**Table 1:** Themes and subthemes from thematic analysis, with accompanying sample tweets

Themes and subthemes	Sample tweets
**Theme 1: Scarcity of liver donors**	
Crowdsourcing for donors	“In need of O + liver donor for a very close family member. If anyone could help Or provide some useful info about liver transplant. Will B very grateful Kindly reshare if possible.!! #Help #livertransplant #liverdonor” “#LiverDonorNeeded A friend’s dad is in urgent need of a Liver donor with the following requirements: 1. Blood Group: O + or O- 2. Age: Between 18–50 years 3. BMI under 27 (1/2) #Hyderabad #Liverdonor”
Desperation to find a donor	“URGENT SOS #LiverDonor required. See the pic for more details. Spread the word. #LiverDonor #Delhi #GangaramHospital #livertransplant #LiverTwitter #MedTwitter #UrgentHelp” “Urgent need of a liver donor with A + ve or O + ve group for a young guy who is on ventilator in ICU in ILBS Vasant Kunj Delhi. #liverdonor #OrganDonation”
Desire to save friend or loved one	“Guys please do help him…. He is my friend #liverdonor #doctor #help #viral” “Ajit is a loving father & grandfather and a kind, funny and generous man. His grandkids deserve many more years to make memories with their beloved grandpa. If you are from #Toronto & have type O+/O- blood, please consider becoming a #liverdonor & giving Ajit the gift of life.”
**Theme 2: Life of a liver donor post-transplant**	
Returning to normal activities	“Better day by day. Thanks for all the prayers. Got a hall pass 4 some real food. #RamNation #liverdonor” “6 months today #liverdonor #officiallyallowedtodrink #donatelife #december4th #6monthanniversary”
Surgical scar as a physical reminder of donation	“Proud of them scars! #liverdonor #selflove #organdonor” “I’m #GivingProof….who says I’m #LiverDonor Only medical documents… or the scar on my belly….”
Exercise as a marker of recovery	“Don’t wait for it Work for it #fitindia #organdonor #LiverDonor. #fitness” “Set you goals #fitnesschallange #HumFitTohIndiaFit #liverdonor #retweet #organdonors”

The themes and subthemes are explained in the following.

### Theme 1: scarcity of liver donors

#### Subtheme 1.1 crowdsourcing for donors

Given the tremendous burden of liver failure on public health, the continued shortage of liver donors and the far reach of social media networks, crowdsourcing for potential donors was not unexpected. “The patient mentioned here is a dear friend and is battling life. Please retweet this to amplify as much reach as possible” exemplified the use of Twitter (X) to communicate the need for a living organ donor beyond one’s immediate social network.

#### Subtheme 1.2 desperation to find a donor

Driving the efforts to crowdsource for a potential donor was probably feelings of desperation as individuals saw their loved ones in a very ill state, “Pleasanton Dad Fighting For Life Needs Live #LiverDonor.” The need for a living donor came across as dire and critical.

#### Subtheme 1.3 desire to save friend or loved one

An important motivation in deciding to become a liver donor was the desire to save a close friend or loved ones, “I wanna be a liver donor for my niece! #supportforpaisley #liverdonor.”

### Theme 2: life of liver donors post-transplant

#### Subtheme 2.1: returning to normal activities

Both liver donors and transplant recipients appeared to experience some ailments post-surgery and needed time to recover and return to normal daily activities, “If you’re a #liverdonor who’s experienced complications, you’re not alone.”

#### Subtheme 2.2: surgical scar as a physical reminder of donation

Interestingly, living liver donors may see their postoperative incision scars with satisfaction and view them as a physical reminder of their act of organ donation, “Sporting my scars in the sun #damnyourbeautystandards #scars #livingdonor #donatelife #liverdonor #organdonor #earnedthem.”

#### Subtheme 2.3: exercise as a marker of recovery

In contrast to the idea of sickness is fitness, which may explain the perception of exercise as a marker of recovery post-transplant, “We’re our own worst enemy. You doubt yourself more. If you can get past that, you can be successful. -Michael Strahan #LiverDonor #fitindia #organdonor. #fitness #fitnessmotivation #fitnessmodel #fitnessfreaks #fitnesslife #fitnessjourney #gymmotivation #gymshark.”

## Discussion

Through a reflexive thematic analysis of tweets, this study revealed several pertinent themes and subthemes regarding the contemporary viewpoints on living liver donation. Given the global shortage of both deceased and living liver donors and persistent system gaps, patients face particularly lengthy wait times, and several demise while waiting for a suitable donor [4]. This is likely a source of frustration, grief and desperation for patients and their family members, who are increasingly turning to social media to appeal for potential donors [14, 15]. The inexpensive and accessible nature of Twitter (X), coupled with its broad user demographics, make it a popular option for patients and families to crowdsource for potential donors. Moreover, soliciting for potential donors over such social media platforms may also be less emotionally taxing than traditional forms of public solicitation e.g. face-to-face communication [21]. 

There are some successes to such social media appeals as evidenced by a report from the National University Centre for Organ Transplantation in Singapore, which recorded seven living liver donor transplants from members of public, not directly related to the patient, performed between 2014 and 2019 [22]. This stands in stark contrast to merely two such procedures in the preceding 18 years. Liver donations from living donors are difficult to come by, and it is probably even rarer for the willing donor to be a stranger. While further published evidence on the effectiveness of social media use for living donor-recipient matching is lacking, there are undisputable anecdotal reports of successes for people who have reached out on social media [23]. The search for a compatible donor may be facilitated by the connectivity and broad dissemination enabled by modern social networks. Nonetheless, there are practical and ethical concerns when using a social media platform like Twitter (X) for living organ donor appeals, including the violation of privacy when broadcasting medical need, lack of supervision and guidelines over posts, inability to verify authenticity of information, potential inequities and uneven agency among patients and families [24]. Similarly, a systematic review highlighted the impact of social media on donor behaviours and the ethical considerations associated with leveraging these platforms for health-related crowdsourcing, underscoring the importance of transparency and trust in these digital engagements [25]. We also identified a few tweets that seemed to advertise the commercial trade of organs, “kidney liver Donor #KidneyDonor BLood type A + #DonorGinjaL_A + #LiverDonor Name: [redacted] Age: 30 years Height: 168 cm Weight: 58kg Blood Type: A + No Phone WhatsApp [redacted] From: INDONESIA.” Illegal organ trafficking is a growing problem in Asian countries [26]. Similar advertisements can be found on Google answers and on Facebook [27]. Many countries prohibit commercial transplantation and only allow blood relatives to act as donors, and transplant donations must be pre-approved by a hospital transplant committee [28]. 

A live liver donation is life-changing especially to patients with advanced cirrhosis and in critical medical condition. A living liver donor is a person who voluntarily donates a portion of their liver to someone in need of a transplant. The liver has the ability to regenerate, allowing both the donor and transplant recipient to have functional livers after the procedure. This is the public messaging commonly adopted by organizations and campaign efforts [29], which aims to highlight the beautiful gift of life bestowed by a liver donor. This sentiment is also common to the tweets by users over Twitter (X).

Another key theme embedded in the contents of the tweets is that of the experiences of living donors post-surgery. As live liver donation is a major surgery after all, donors would need time to recover – they should be able to perform most normal activities within a month – and do more strenuous activities two to three months post-surgery, before recovering to preoperative health status around six months out [30]. As such, live liver donors may need several weeks or months to fully recover from the surgery, during which they may have to take time off from work or other activities. Although it was not always apparent whether the tweets were referring to live donors or transplant recipients, there were mentions of the various physical challenges faced by liver donors when returning to the normal activities of daily living, e.g. “I miss being able to cough without the feeling that my abdomen is ripping apart and my insides are falling out. #surgerythings #liverdonor.” The mistaken notion that donors are healthy persons may overlook the pain, stress and the higher morbidity risks (e.g. chronic pain, increased risk of liver disease and increased risk of reduced liver function) they experience post-surgery [31]. It is therefore important for physicians to counsel potential live liver donors of the risks associated with the surgery and continue to follow them up to ensure that they receive adequate support and resources post-surgery.

Closely linked to the idea of fitness is perhaps physical exercise, which may also be associated with a greater sense of normalcy as one’s physical capability is a measurable way of benchmarking against one’s original physical state [32]. For liver donors, physical activity could represent another element of normality post-surgery, while for transplant recipients, engaging in physical exercise may be a form of self-care and meaningfully making the most of their new lease of life. As organ donations are often thought to be a gift of life, based on the findings by Wiltshire et al., organ transplant recipients regarded staying fit and active as a way of displaying gratitude to their donors and gaining control over their future health [32]. 

Part of the return to normalcy post-surgery could also involve getting accustomed to the large surgical scar left from the procedure. A previous qualitative study highlighted the uniqueness of scarring in the living donor population as it is the result of a planned, elective surgery for an otherwise healthy individual [33]. In contrast to conventional beliefs about cosmetic issues relating to a prominent physical scar (e.g. embarrassment, depression and negative self-esteem), living donors seem to wear their scars as a symbol of pride. These were reflected in tweets found in the present study, “Proud of them scars! #liverdonor #selflove #organdonor”, and they could stem from their positive feelings for having saved a life and positive thoughts about overcoming the tiresome process of liver donation. In a qualitative study of 26 anonymous live liver donors, there was no mention of any body image or cosmetic concerns and instead, discussions about the negative aspects of scarring pertained only to the possible threat to their desired anonymity [33]. 

Collectively, the public conversations over Twitter (X) reflect (1) the continued shortage of liver donors and (2) the experiences of liver donors post-surgery. They provide an inkling of the experiences of individuals at various stages of living liver transplantation process, and they highlight the need for further research into how we could encourage further donation and better support donors post-procedure. In particular, there is a lack of a clear understanding of the priorities considered important to a living liver donor. Live liver donation is a complex procedure that requires careful evaluation of the donor’s health and the compatibility of the donor and transplant recipient. In addition to the work that goes into a thorough perioperative assessment, more should be done for donors post-procedure as well. This could include enhancing follow-up care, providing psychological support, and creating support networks for donors. These recommendations aim to address the needs and challenges faced by liver donors post-surgery.

Improving liver donation rates requires a multi-faceted approach that addresses the various barriers to donation. Applying the HPT framework [17], the study uncovered crucial themes in the public conversation about liver donation, primarily focusing on the acute scarcity of liver donors and the nuanced experiences of donors after the transplant (Content). These themes are not mere reflections of individual sentiments but represent significant policy concerns. Political and administrative factors play a role in shaping the success of health policies, particularly those related to organ donation. Governmental support and legislative frameworks can either facilitate or hinder organ donation efforts [34]. Policies that streamline the donation process, provide clear guidelines, and offer legal protection to donors and recipients can encourage participation. Administrative efficiency, including the management of donor registries and transplant coordination, significantly impacts the effectiveness of these policies. Political will and commitment from policymakers to prioritize organ donation in the health agenda are also probably crucial for sustaining and enhancing these initiatives.

The study findings emphasize the need for concerted efforts to address donor shortages and improve post-donation care, suggesting a potential recalibration of existing health policies and strategies in this domain. The sociocultural backdrop plays a pivotal role in shaping the discourse around liver donation (Context). Cultural beliefs and social norms intertwine with attitudes towards organ donation, creating a complex tapestry of public opinion. Although we were unable to reliably extract detailed demographic information from Twitter (X) bios or tweets alone, especially after the recent API changes, it is known that in some societies, beliefs about bodily integrity after death, spiritual concerns, and family decision-making processes can affect individuals’ willingness to donate organs [35]. Social norms regarding altruism, community support, and collective well-being can influence organ donation rates. Health policies must navigate these delicate cultural dynamics to foster a supportive environment for organ donation. Tailored communication strategies that respect and address cultural sensitivities would help build trust and encourage participation in organ donation programs [36]. 

Additionally, economic and political factors, such as healthcare policies and organ donation laws, add layers of complexity to how liver donation is perceived and practiced. Our findings also point to the influential role of technology, particularly social media platforms like Twitter (X), in crowdsourcing for potential donors. The themes emerging from Twitter (X) conversations can also be instrumental in initiating policy dialogue. The discourse on Twitter (X) can be seen as part of a feedback loop in policy development. As policies are implemented (or not), their impact and public reception are often reflected in social media discourse. The tweets bring to light ethical and practical considerations in liver donation – from concerns about donor safety and well-being to issues of equity in organ allocation (Process). These considerations are critical for developing comprehensive, ethical, and practical policies that address the complex realities of liver donation. Finally, patients, their family members, policymakers and advocacy groups (Actors) emerge as crucial actors for any policy reform process, based on the insights gleaned from these online discussions.

### Limitations of this study

First, the findings of this exploratory analysis may not be entirely generalizable as the majority of Twitter (X) users are from the North American regions [37]. In particular, there may be certain cultural differences between Asian and Western countries [38]. It would be ideal to supplement the analysis with further demographic, educational, and cultural characteristics to enrich the findings. The analysis may also be triangulated with data from other social media platforms or forums to give a more comprehensive understanding of the public conversation worldwide. Using Twitter (X) as the sole data source has its advantages and disadvantages. The platform has been frequently used by academics as it excels in the rapid dissemination of information, allowing users to share and respond in real-time to events and topics [12, 13], and it is also known for its openness, with many posts being publicly accessible without restrictions, as compared to Facebook or Instagram where content and posts are frequently shared within private or closed groups [39]. Second, as there is no predefined glossary of search terms for liver donation over Twitter (X), this means that certain sentiments may not have been picked up using our present search strategy and it may have introduced some selection bias. Last but not least, as tweets have a short character limit and it was not always apparent whether the tweets were referring to live donors or transplant recipients, certain interpretations may have been mistaken as a result. To address these challenges, future research could analyse reply chains to capture the broader context of conversations. By examining interactions and follow-up comments, researchers could gather additional details that clarify or elaborate on initial tweets. Additionally, reviewing the bios of tweeters, when available, could provide valuable insights into the background and potential biases of users. This extra context would help in interpreting tweets by understanding the perspectives and motivations of the individuals behind them. However, it is important to note that not all tweets are part of reply chains, and not all users have detailed bios, which may still leave some tweets open to interpretation.

## Conclusion

In conclusion, this study qualitatively analysed a large corpus of tweets and highlighted themes relevant to the weighty issue of living liver donation. The findings, as represented by Twitter (X) users, affirm both the continued shortage of liver donors and the uniqueness of the living donor experience. When viewed through this lens, more needs to be done to increase the awareness of the journey each liver donor undertakes and hopefully, the number of donors as well. With the growing use of social media to crowdsource for donors, guidelines and advisory should also be developed for related posts.

## Data Availability

The data that support the findings of this study are available from the corresponding author upon reasonable request.
